# Competence by Design: The Role of High-Stakes Examinations in a Competence Based Medical Education System

**DOI:** 10.5334/pme.965

**Published:** 2024-02-06

**Authors:** Farhan Bhanji, Viren Naik, Amanda Skoll, Richard Pittini, Vijay John Daniels, C. Maria Bacchus, Glen Bandiera

**Affiliations:** 1 Royal College of Physicians and Surgeons of Canada, Ottawa, ON, Canada; 2 Faculty of Medicine and Health Sciences, McGill University, Montreal, QC, Canada; 3 Medical Council of Canada, Ottawa, ON, Canada; 4 Department of Anesthesiology and Pain Medicine, University of Ottawa, Ottawa, ON, Canada; 5 Department of Obstetrics and Gynecology, University of British Columbia, Vancouver, BC, Canada; 6 Temerty Faculty of Medicine, University of Toronto, Toronto, ON, Canada; 7 Division of General Internal Medicine, University of Alberta, Edmonton, AB, Canada; 8 General Internal Medicine Subspecialty Exam Board Chair, Royal College of Physicians and Surgeons of Canada, Ottawa, ON, Canada; 9 Department of Medicine, University of Calgary, Calgary, AB, Canada; 10 University of Toronto Temerty Faculty of Medicine, Executive Director, Standards and Assessment, Royal College of Physicians and Surgeons of Canada, Canada

## Abstract

Competency based medical education is developed utilizing a program of assessment that ideally supports learners to reflect on their knowledge and skills, allows them to exercise a growth mindset that prepares them for coaching and eventual lifelong learning, and can support important progression and certification decisions. Examinations can serve as an important anchor to that program of assessment, particularly when considering their strength as an independent, third-party assessment with evidence that they can predict future physician performance and patient outcomes. This paper describes the aims of the *Royal College of Physicians and Surgeons of Canada’s* (“the Royal College”) certification examinations, their future role, and how they relate to the Competence by Design model, particularly as the culture of workplace assessment and the evidence for validity evolves. For example, high-stakes examinations are stressful to candidates and focus learners on exam preparation rather than clinical learning opportunities, particularly when they should be developing greater autonomy. In response, the Royal College moved the written examination earlier in training and created an exam quality review, by a specialist uninvolved in development, to review the exam for clarity and relevance. While learners are likely to continue to focus on the examination as an important hurdle to overcome, they will be preparing earlier in training, allowing them the opportunity to be more present and refine their knowledge when discussing clinical cases with supervisors in the Transition to Practice phase. The quality review process better aligns the exam to clinical practice and can improve the educational impact of the examination preparation process.

## Introduction

What is the role of high-stakes examinations in the era of competency based medical education (CBME)? CBME and programmatic assessment represent a shift in assessment philosophy and design to frequent low-stakes assessments of trainees in their daily work, and some have suggested that this should signal the end of examinations. Unfortunately, health professions education continues to exhibit a “‘failure to fail” culture [1,2] for trainees in the clinical environment and we cannot assume this will change with CBME until we determine the validity of workplace-based assessment. While most trainees succeed and many excel in postgraduate medical training, any clinical faculty who engage in honest reflection will almost certainly identify trainees about whom they have concerns — aspiring physicians whom they would not trust to look after their own family members by the end of their clinical training. These concerns may have to do not only with the trainees’ medical expertise but may also relate to other core competencies such as communication, collaboration, or professional conduct. CBME, including the Competence by Design (CBD) model of the Royal College of Physician and Surgeons of Canada (hereafter referred to as the Royal College) [3], has tremendous potential to reshape education, support learning, and promote a growth mindset in our future colleagues. For this opportunity to be realized, there needs to be a cultural shift wherein learners recognize a safe, supportive learning environment that allows acknowledgment of one’s limits and candid discussion of areas for improvement. Layered on top of that, there needs to be increased accountability of front-line faculty and Competence Committee members to determine trainees’ progression toward unsupervised practice. A well-designed program of assessment [4] that emphasizes frequent assessment at the top of Miller’s pyramid [5], that is, what a trainee “does” in clinical practice (related to all CanMEDS [6] Roles), offers the theoretic potential to address the prevalent failure to fail.

There are a myriad of factors underpinning the failure to fail phenomenon, and CBD is not likely to address all of them without meaningful culture change. At the learner level, trainees may select easy cases where they perform well and/or seek to have more lenient faculty supervisors assess their competence because of the perceived stakes of the assessment and its impact on their progression. This both hinders development of a growth mindset and is a threat to the validity of the eventual summative assessment. At the clinical supervisor level, the expectations of performance may be unclear and (untrained) raters may demonstrate considerable variability. Leniency bias is also a concern, especially in contexts in which there is a social interaction with trainees and ones in which raters may attribute poor performance to the circumstance or case, choosing not to document their concerns because they assume that the poor performance they observed was a one-time occurrence. At the program level, there may be concerns that “failure” of trainees reflects poorly on the training program, that there awaits a substantial remediation workload once failure has been identified, and that failing trainees can leave gaps in clinical service delivery schedules. External factors may also influence trustworthy assessment decisions such as the threat of litigation from underperforming trainees who are not progressing as expected. These factors, among others, coupled with a robust examination process, have perpetuated an environment where some programs implicitly rely on national Royal College examinations to identify poorly performing candidates and create a barrier to these candidates entering unsupervised practice.

Although the utility and role of summative certification examinations conducted by national certifying bodies have been questioned particularly during the early phase of the COVID-19 pandemic [7], the literature suggests that certifying/licensure examinations help to predict future physician performance as well as important patient outcomes [8,9,10,11,12,13,14,15,16]. They can serve as important safeguards for the public, especially when a local training program either cannot identify a trainee struggling or is struggling with the aforementioned failure to fail. In Canada, trainees at risk of failing the Royal College examination are often identifiable relatively early in training [17], but this “signal” is not translated into documented, usable assessment data. Those with the weakest in-training evaluations have a four-fold risk of failing the Royal College Internal Medicine examination [18], yet such individuals are still considered by their program to be eligible for the examination. Since Royal College examinations are developed with the goal of determining competence for unsupervised practice, it can be argued that not all training programs are meeting their obligation in the social contract [19] to complete honest assessments of learners throughout their training and to make accurate decisions about competence and program completion. National summative examinations, therefore, continue to serve an important complementary role in the certification process.

Each Specialty and Subspecialty of the Royal College has an Examination Board that is responsible for the development of its examinations. This Board reports to a national Specialty Committee, that develops the educational standards, as well as to an overarching Royal College Examination Committee that develops policy, and provides guidance for, as well as oversight of, the quality and validity of the examination. The membership for the Examination Committee represents leaders in assessment from across Canada, including current and past Exam Board Chairs that represent a variety of surgical, medical and lab-based disciplines, as well as a PhD scientist focused on assessment. This process is supported by an internal Exam Quality and Analytics unit, that includes a Psychometrics team, and an Operations team at the Royal College that implements the delivery of the examinations. In total, over 1,300 specialist physicians volunteer their time, and close 50 fulltime staff, contribute to the examination process across our 67 Specialties/Subspecialties.

This paper, authored by leaders within the Examination Committee and staff physicians with leadership roles in examinations and certification, describes the aims of the Royal College high-stakes certification examinations, their future role, and how they relate to the CBD model. Acknowledging that preparation for high-stakes examination, in and of itself, can heavily influence trainee learning and behaviour, we describe how the Royal College examinations are undergoing important modifications to support the educational design of CBD.

## Purpose of Royal College examinations

Royal College examinations aim to provide a fair assessment to all candidates, whether they have trained in a CBD program or outside of that context. The critical first step of all assessment activities should be to determine the purpose of the assessment. Royal College examinations are blueprinted, developed, and delivered with the intention to determine competence for unsupervised clinical practice in Canada, providing a national benchmark for each specialty based on the contemporary scope of practice within each discipline. Most candidates are from Canadian postgraduate training programs; however, a substantial minority are international medical graduates who have completed their medical training outside of Canada and either are now practising in Canada or want to obtain certification (and licensure) in Canada. It is thus an additional function of the examination to ensure that all who seek licensure via certification in the Royal College are assessed against the same standard across the same expected scope of practice of the discipline.

We utilize the mnemonic CARVE as a useful tool to highlight key elements to consider in an assessment activity, like an examination or a program of assessment [20,21]. Based on the Ottawa Consensus Framework for good assessment [22], the mnemonic outlines five key considerations for high-stakes examinations: Cost-effectiveness; Acceptability; Reliability; Validity; and Educational Impact.

The primary aim of the examinations is to utilize a process that is independent from, and at arm’s length to, candidates’ postgraduate medical education or clinical practice supervision, where leniency bias can be a concern. The examination leverages a standardized process employing trained examiners with content expertise to complement the program of assessment in the workplace, which also forms an important part of the certification process. This additional source of assessment (i.e., the examination) can help with promoting the public trust in decision-making around certification. Given that the Royal College does not administer ongoing recertification examinations to physicians in practice, this initial certification carries substantial stakes for both the candidate and society. Although the primary purpose of the Royal College examinations is to determine the competence of candidates, it is well recognized that assessment drives learning, so the educational impact of assessment decisions (e.g., the timing and content of examinations) remains important. As the culture of workplace assessment changes and the evidence of the validity of CBD assessment becomes clearer over time, the Royal College will continue to revise and adapt the examinations while ensuring a robust national standard for certification.

## Nine key design adaptations for examinations in an integrated competency-based national certification process

The Examination Committee of the Royal College has worked closely to incorporate advice or policy from standing Committees, Examination Boards and Clinician Educators of the College to develop these adaptations. As with any complex change in a large system with many stakeholders, consensus may not always be possible, but the Committee did reflect on issues and develop an integrated approach that has allowed Specialty specific Examination Boards to implement the changes. The impact of the changes are reviewed as part of the ongoing Continuous Quality Improvement of the Committee’s work.

The COVID-19 pandemic has required considerable change to the examination process, with a move away from paper-and-pencil examinations and face-to-face oral and OSCE examinations to the use of online platforms for both. The adaptations outlined below enabled the Royal College to maintain rigour and validity in the examination process despite the change to the methods of delivery and the specific examination structure.

### Earlier timing for Examinations

Trainees, even those in CBD programs, are likely to continue to study hard for their certifying examinations. The opportunity to move the examination earlier in training allows trainees to demonstrate their competence on the exam and then to focus their learning on their clinical education for their final few months of training. That opportunity, coupled with the strong theoretical understanding of the discipline through examination preparation, allows learners to amplify their learning in the Transition to Practice (TTP) phase (the final stage of CBD programs), a commonly underemphasized aspect of training and a key opportunity to rectify often-identified perceived lack of readiness for practice in graduating trainees across disciplines. For the trainee who is not successful at their examination, the earlier examination timing allows them an opportunity for remediation and examination preparation support while they still are registered in the training program. To date, the Royal College written examinations have been moved from the end of training into the Core of Discipline stage, the third of the four stages of CBD programs [3,23]. Thus far, the anecdotal feedback has been positive in terms of learner knowledge leading in to the TTP stage, and candidates are able to pass the examination at similar rates to historical cohorts that came to the examination later. The workload to produce an additional earlier high-quality examination, both in terms of content development from specialist physicians, and operational support is substantial.

### Alignment across assessments

All Royal College examinations undergo review and/or revisions to their blueprints to ensure they reflect clinical practice in Canada. Examination boards consider what is best assessed on examinations and conversely what is best determined through workplace-based assessment, supporting a complementary approach to the two forms of assessment in the certification process. The approach does not require that topics be covered exclusively in one form of assessment or the other and there can, and should, be overlap for the examination to function as the external checkpoint for certification for both trainees from Canadian programs and international medical graduates who are not from CBD training programs. The principle is to consider the optimal approach for the competencies being assessed. For example, professional conduct is best assessed in the clinical environment, whereas the candidate’s management of a less common but important clinical condition, the type that defines the competent specialist in the domain, may be better assessed on the examination. Simulation based assessment, that occurs within training programs, but is nationally developed, has also been incorporated into certification process when relevant to a Specialty [24]. Where the certification process includes both a written and an applied examination (oral, objective structured clinical examination [OSCE], or practical), the examination board also determines what is best assessed on each examination.

### Sequencing of examinations

Royal College examinations are transitioning toward a written-before-applied format where they are blueprinted and designed such that a candidate must pass the written examination before they can present to the applied examination. This transition has already occurred for the Royal College’s multiple-choice examinations, and it is being implemented for examinations with short-answer questions as the specialties with those examinations transition to CBD.

### Transparency

Transparency is being improved so that candidates understand the examination process, the blueprint, and the sample questions related to the examination. This has required extensive work from all the Royal College’s examination board chairs and their executives, as well as substantial updates to the Royal College’s website. These efforts to increase transparency help to reduce construct-irrelevant variance so variation in scores is more related to true differences in competence.

### Development of examiners and exam leadership

Enhanced training of examiners and examination board leadership is being offered to help them to fulfill the duties of their role and to consider competence in the Canadian context as the target of the examination. Examiners aim to develop questions such that a competent specialist or subspecialist would be able to succeed on the examination. While some questions may be more difficult than others, the overall examination is built to consider competence as the target. The Royal College is fortunate to have over 1,300 examination board members across 67 specialties and subspecialties who volunteer their time to ensure the standards of their specialty are maintained in the certification process. These examiners are supported by an operational examination development and delivery team, as well as a psychometrics and data analytics team who provide education and ongoing support to board chairs, vice-chairs, executives, and board members as they develop and review their examinations.

### Quality review of the examination

An examination quality reviewer who does not participate in the question development process reviews questions for clarity and relevance to the specialty or subspecialty. This process adds to the validity of the examination and helps focus the examination on the most relevant material. It also may support the educational impact by focusing learners on relevant clinical practice.

### Global rating scales

For specialties and subspecialties that utilize an applied examination (an oral, OSCE, or practical examination), the scoring tool has moved away from checklists to a global rating scale focusing on higher order thinking that better aligns with physician tasks such as clinical decision-making rather than testing rote memory. This approach aligns better with the determination of competence at the level of a certification examination and rewards more thoughtful answers from candidates over the completeness that may be more appropriate at the level of more junior learners.

### Updating the psychometric approach

Updated psychometric processes are being implemented to align with more competency-based approaches. For example, the Royal College moved to theta as the primary reliability measure (more related to decision consistency of the examination and more aligned with a criterion-referenced approach to the examination) instead of Cronbach’s alpha, which is dependent on the cohort of candidates taking the test (and more dependent on achieving a spread of scores, which aligns more with a norm-referenced approach). Additionally, there is an enhanced post-examination question review and psychometric process to identify and consider questions that may not be working as intended, which may need to be removed from the examination, as well as an enhanced process for conducting standard setting with panellists from the respective examination boards.

### Quality improvement

Continuous quality improvement from one iteration of an examination to the next is important to any organization involved in high-stakes assessment. The Royal College’s continuous quality improvement process is supported by the review of candidates’ post-examination surveys, the assessments of examination quality reviewers, psychometric data, and a review of the specialty-specific examination by the overarching Royal College Examination Committee.

## Summary

Examinations continue to play an integral role in the Royal College certification process as an independent, third-party assessment that complements workplace-based assessment. They provide a credible anchor to the program of assessment within CBD and allow candidates from both CBD and non-CBD training programs to be assessed against a common standard. Only through careful development and delivery can the Royal College maintain the validity of its examination process. Although the Royal College is developing more robust programs of assessment, including more frequent workplace-based assessment, changes in the certifying examination need to be considered carefully to maintain public trust and ensure validity in the Royal College’s entire process of assessment. The stepwise approach to examination reform that has been undertaken in Canada provides aggregate data accumulated from the examinations that may inform the program evaluation of the move to CBD. Changes in trainee performance on the examination, particularly if there is improvement in examination scores or pass rates, or even pass rates that are similar to historical controls but with the examination taking place months to a year earlier, can serve as a signal to a positive educational effect of CBD.

## Disclaimer

The views and opinions expressed in this article are those of the authors and do not necessarily reflect the official policy or position of the Royal College of Physicians and Surgeons of Canada (“Royal College”). Information in this article about Competence by Design (“CBD”), its implementation and related policies and procedures do not necessarily reflect the current standards, policies and practices of the Royal College. Please refer to the Royal College website for current information.
